# Comparative whole-genome analyses of selection marker–free rice-based cholera toxin B-subunit vaccine lines and wild-type lines

**DOI:** 10.1186/s12864-015-1285-y

**Published:** 2015-02-05

**Authors:** Koji Kashima, Mio Mejima, Shiho Kurokawa, Masaharu Kuroda, Hiroshi Kiyono, Yoshikazu Yuki

**Affiliations:** Division of Mucosal Immunology, Department of Microbiology and Immunology, The Institute of Medical Science, The University of Tokyo, Tokyo, Japan; Asahi Kogyosha Co., Ltd., Tokyo, Japan; Crop Development Division, NARO Agriculture Research Center, Niigata, Japan; International Research and Development Center for Mucosal Vaccines, The Institute of Medical Science, The University of Tokyo, Tokyo, Japan

**Keywords:** Plant-made pharmaceuticals, Oral vaccine, Whole-genome resequencing, Transgenic rice, MucoRice-CTB, Variant comparison

## Abstract

**Background:**

We have developed a rice-based oral cholera vaccine named MucoRice-CTB (Cholera Toxin B-subunit) by using an *Agrobacterium tumefaciens*–mediated co-transformation system. To assess the genome-wide effects of this system on the rice genome, we compared the genomes of three selection marker–free MucoRice-CTB lines with those of two wild-type rice lines (*Oryza sativa* L. cv. Nipponbare). Mutation profiles of the transgenic and wild-type genomes were examined by next-generation sequencing (NGS).

**Results:**

Using paired-end short-read sequencing, a total of more than 300 million reads for each line were obtained and mapped onto the rice reference genome. The number and distribution of variants were similar in all five lines: the numbers of line-specific variants ranged from 524 to 842 and corresponding mutation rates ranged from 1.41 × 10^−6^ per site to 2.28 × 10^−6^ per site. The frequency of guanine-to-thymine and cytosine-to-adenine transversions was higher in MucoRice-CTB lines than in WT lines. The transition-to-transversion ratio was 1.12 in MucoRice-CTB lines and 1.65 in WT lines. Analysis of variant-sharing profiles showed that the variants common to all five lines were the most abundant, and the numbers of line-specific variant for all lines were similar. The numbers of non-synonymous amino acid substitutions in MucoRice-CTB lines (15 to 21) were slightly higher than those in WT lines (7 or 8), whereas the numbers of frame shifts were similar in all five lines.

**Conclusions:**

We conclude that MucoRice-CTB and WT are almost identical at the genomic level and that genome-wide effects caused by the *Agrobacterium*-mediated transformation system for marker-free MucoRice-CTB lines were slight. The comparative whole-genome analyses between MucoRice-CTB and WT lines using NGS provides a reliable estimate of genome-wide differences. A similar approach may be applicable to other transgenic rice plants generated by using this *Agrobacterium*-mediated transformation system.

**Electronic supplementary material:**

The online version of this article (doi:10.1186/s12864-015-1285-y) contains supplementary material, which is available to authorized users.

## Background

Production of pharmaceutical ingredients by using plant expression systems (plant-made pharmaceuticals, or PMPs) has become a promising technology [1,2]. The advantages of producing PMPs compared to conventional production systems (such as large-scale bacterial fermentation) are as follows: cost-effectiveness, adaptability for scaling up and possibility to produce eukaryotic proteins with correct 3-dimensional structures [1,3]. Plant-based systems such as transient expression or transgenic systems developed during the last two decades have been reviewed by Paul and Ma [4]. In both systems, higher protein yield has been achieved through improvement of the expression vectors [4,5]. Despite intensive efforts aimed at PMP marketing, only glucocerebrosidase produced in plant cell culture for treatment of Gaucher’s disease has been approved for human use [6].

We previously reported MucoRice-CTB, transgenic rice expressing cholera toxin B-subunit (CTB) designed as an oral vaccine against cholera [7]. MucoRice provides a suitable vehicle for expression, accumulation, and mucosal delivery of antigens that are not only stable at room temperature for several years without loss of immunogenicity, but are also protected from digestive enzymes in the gastrointestinal tract. Oral vaccination of mice and macaques with MucoRice-CTB resulted in the induction of antigen-specific serum IgG and mucosal IgA responses with toxin-neutralizing immunity [8]. Because of sequence similarity between cholera toxin (CT) and heat-labile enterotoxin from enterotoxigenic *Escherichia coli*, MucoRice-CTB successfully induced protective immunity against both *Vibrio cholerae*–induced and enterotoxigenic *E. coli–*induced diarrhea [9]. We also achieved high-yield CTB production in rice seeds by using a CTB overexpression system together with an RNA interference (RNAi) cassette to suppress the production of major endogenous storage proteins, prolamin 13 kDa and glutelin A. The amount of CTB produced in rice endosperm without RNAi reached only 1/6 of that of MucoRice-CTB with RNAi [10].

To perform a phase I study as the first step towards human application of MucoRice-CTB, we have recently established a selection marker–free line (51A) as a seed bank by using co-transformation with two different *Agrobacterium tumefaciens* strains*,* each carrying a distinct T-DNA containing either a selection marker cassette or the CTB and RNAi cassettes [11]. This *Agrobacterium*-mediated transformation system includes several steps: (1) sterilizing Nipponbare seeds with sodium hypochlorite solution, (2) induction of calli with plant hormones, (3) transformation with *Agrobacterium* carrying T-DNA, (4) regeneration in the presence of plant hormones followed by cultivation under antibiotic pressure, (5) propagation of the three MucoRice-CTB lines for at least five generations by self-pollination to fix the desired transgene. Since it has been reported that the *Agrobacterium*-mediated transformation system may cause genomic changes in the host organisms [12,13], it is essential to assess the effects of our *Agrobacterium*-mediated transformation system on the genome of MucoRice-CTB seed bank intended for human use.

Recently, next-generation sequencing (NGS) has greatly influenced the discovery of genetic markers [14] and facilitated transcriptomic approaches [15] in various organisms. Furthermore, the increasing availability of reference genomes has promoted resequencing in a wider variety of species. Resequencing allows detecting substantial numbers of genomic variations including single-nucleotide polymorphisms (SNPs) and insertions and deletions (InDels) between the target and reference genomes [16]. In addition to revealing the differences between rice subspecies (*japonica* and *indica*), resequencing analysis has provided insights into the diversity of domesticated rice [17-19].

In this study, using NGS, we investigated the genomic differences between three selection marker–free MucoRice-CTB lines, including the line 51A intended for phase I clinical trial, and two wild-type (WT) rice lines (*Oryza sativa* L. cv. Nipponbare from two different sources). The three MucoRice-CTB lines were selected by the level of CTB protein production and elimination of the marker gene used for the initial transformant selection [11]. We found that these MucoRice-CTB and WT lines are almost identical at the genomic level. The type of comparative analysis reported here can be used to estimate genome alterations not only in MucoRice-CTB but also in other transgenic rice plants generated by using a similar *Agrobacterium*-mediated transformation system.

## Results

### Read alignment to the rice reference genome

Genomic DNAs of five rice lines (three marker-free MucoRice-CTB lines, 50A, 51A, and 55A; two Nipponbare WT lines, WT1 and WT2) were sequenced by NGS. After filtering to exclude reads with low sequence-quality scores, more than 300 million paired-end reads were obtained for each line (Table 1). The reads from each line were aligned separately to the rice reference genome [20,21]. In addition to the 12 rice chromosomes, nucleotide sequences of the CTB expression construct, hygromycin resistance gene (hygromycin phosphotransferase: HPT) used as a selection marker, and the binary vector used for *Agrobacterium*-mediated transformation were added to the reference to examine whether these sequences are integrated into the genomes. The resulting mapping rates ranged from 97.1 to 98.1% (Table 1). In all results of mapping of MucoRice-CTBs, we confirmed that the HPT gene had been segregated and excised during the passage of generations (Additional file 1: Figure S1). The coverage rate ranged from 99.1 to 99.6%, whereas the depth (the average number of reads covering a genome) ranged from 84.3 to 101.3 (Table 1).

**Table 1:** **Summary of sequence reads for each line**

**Line**	**Total reads**	**Mapped reads**	**Mapping rate (%)**	**Genome coverage (bp)**	**Coverage rate (%)**	**Depth (fold)**
50A	384,384,291	375,891,543	97.8	371,762,264	99.6	101.3
51A	362,591,974	352,782,360	97.3	371,570,800	99.6	95.1
55A	356,620,373	349,808,296	98.1	369,691,614	99.0	94.7
WT1	342,044,404	332,246,878	97.1	371,589,770	99.6	89.4
WT2	323,436,829	314,249,500	97.2	371,575,970	99.6	84.3

Mapping rate represents the ratio of the number of mapped reads to that of total reads. Covered length represents the number of genome bases covered with at least one read. Coverage rate is the ratio of covered length to the total length of the rice reference genome (373,245,519 bps, IRGSP-1.0, build 5 [21]). Depth was calculated by dividing the total length of all mapped reads (100 bps for each read) by covered length.

### Variant calling and distribution

**Figure 1:**
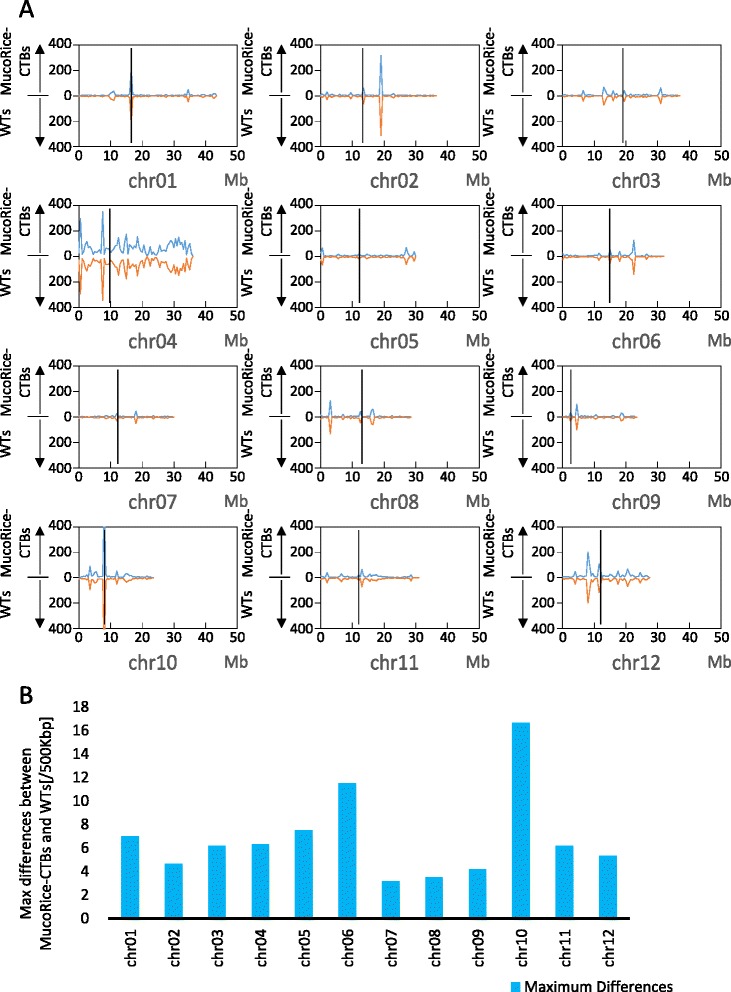
**Variant distribution profiles over the 12 chromosomes of MucoRice-CTB and WT rice lines. (A)** Variant distribution calculated in consecutive non-overlapping 500-kbp windows was averaged for three MucoRice-CTB lines or two WT lines. Blue lines in the upper half of each graph represent average distributions for MucoRice-CTB lines, orange lines in the bottom half represent average distributions for WT lines. The vertical axis represents the number of variants; the values increase upward for MucoRice-CTB lines and downward for WT lines. Black vertical lines in each graph indicate centromere positions. **(B)** The maximum differences in the numbers of variants per 500-kbp window between MucoRice-CTB and WT lines on each chromosome.

The total numbers of detected variants (SNPs and InDels) ranged from 19,103 to 20,623 in the examined lines (Table 2). Variant distribution profiles were calculated in non-overlapping, consecutive 500-kbp windows for each chromosome, and the averages for MucoRice-CTBs and WTs were compared (Figure 1A). On chromosomes 1, 2, 4, 10, and 12, regions with higher variant density than other regions on the same chromosomes were observed. On chromosomes 1 and 10, these regions located close to the centromeres. Variant distribution showed substantial consistency over most genome regions in MucoRice-CTBs and WTs, since we observed the variant densities varied from 0 (e.g., between 9 and 9.5 Mbp on chromosome 1 in MucoRice-CTBs and WTs) to 648.0 (between 8 and 8.5 Mbp on chromosome 10 in WT lines), whereas the differences for every corresponding 500-kbp windows throughout the genome of MucoRice-CTBs and WTs were at most 16.7 on chromosome 10 (Figure 1B).

**Table 2:** **Distribution of variants among MucoRice-CTB and WT lines**

	**Total**					
		**Variant consistency, quality ≥30, covered ≥4 times in all lines**				
			**Line-specific**			
**Line**			**Total (Mutation rate)**	**Ins**	**Del**	**SNPs**
50A	19,802	12,706	798 (2.15 × 10^-6^)	88	222	488
51A	19,612	12,586	619 (1.67 × 10^-6^)	54	224	341
55A	19,398	12,736	842 (2.28 × 10^-6^)	96	263	483
WT1	19,103	12,477	524 (1.41 × 10^-6^)	47	170	307
WT2	20,623	12,927	826 (2.22 × 10^-6^)	79	282	465

Three filters for improving the accuracy of each variant (described in the Methods section) were applied to the total variants (shown as “Total” in the second column). Line-specific variants such as Ins, insertions; Del, deletions and SNPs were selected in accordance with the sharing profile from filtered variants. Mutation rates represent probability of a mutation per nucleotide.

### Comparison of the types of nucleotide substitutions of SNPs

**Figure 2:**
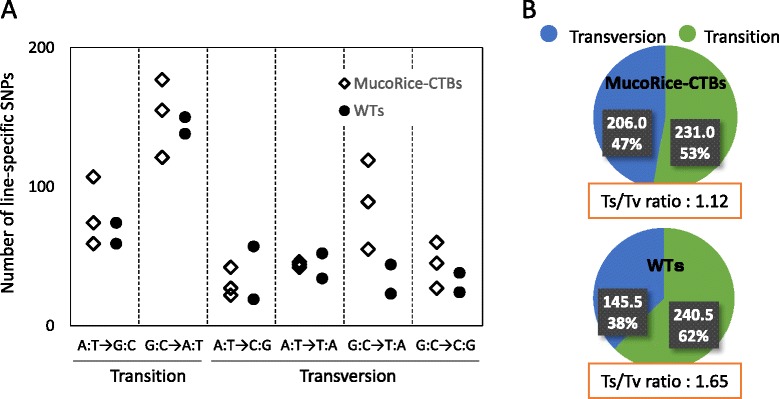
**Line-specific transitions and transversions in MucoRice-CTB and WT. (A)** Numbers of line-specific substitutions in each MucoRice-CTB line and WT line. **(B)** Average numbers and percentage of transitions (Ts) and transversions (Tv), and the Ts/Tv ratio in MucoRice-CTB and WT lines.

Line-specific SNPs were subdivided into transitions (Ts) and transversions (Tv). Nucleotide substitution profiles were obtained for each line (Figure 2A). G to A and C to T were the most frequent Ts in both MucoRice-CTBs and WTs. We also found that the Tv frequency from G to T and C to A was increased only in MucoRice-CTBs. The Ts/Tv ratio for MucoRice-CTBs and WTs were 1.12 and 1.65, respectively (Figure 2B). These results suggest that the substitution patterns in MucoRice-CTBs and WTs were similar.

### Comparison of variant-sharing profiles among MucoRice-CTBs and WTs

**Figure 3:**
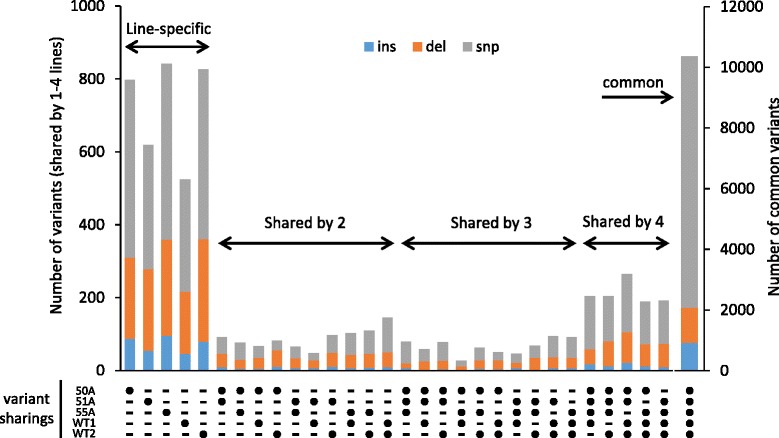
**Variant-sharing profiles.** Number of variants in each MucoRice-CTB or WT line. Dots and dashes at the bottom indicate the presence or the absence of variants, respectively. More than one dot beneath a bar indicates that corresponding lines share the variants. The five leftmost bars show line-specific variants, whereas the rightmost bar shows common variants shared by all lines.

All variants were classified as line-specific (defined as a variant without being shared by other lines), shared by two, three, or four lines, or common to all fivelines (Figure 3). The most abundant variant in number (10,369) was of common type. Since the positions of all variants relative to the reference genome could be determined, we could define candidate line-specific variants by excluding the variants present in more than one line. Mutation rates throughout the genome were calculated by dividing the number of line-specific variants in each line by the covered genome length and ranged from 1.41 × 10^−6^ to 2.28 × 10^−6^ and the average number of line-specific variants was 720 (Table 2). Average numbers of line-specific variants for MucoRice-CTBs or WTs were similar for both totals and breakdowns (insertions, deletions, and SNPs).

### Classification of variants by potential impact on protein function

Using SNPEff software and publicly available rice data sets [20], we predicted the effects of variants on protein function and categorized all of the line-specific variants into 23 effect types (Table 3), which we then grouped into four larger categories (HIGH, MODERATE, LOW or MODIFIER [22]) on the basis of the assumed severity of each effect. Most variants belonged to the MODIFIER category, which is inferred to have only a weak impact. In the HIGH category, 40 out of 47 variants were frame shifts and their numbers were similar among all lines. In the MODERATE category, there were 21 non-synonymous nucleotide changes in the coding regions (which change an amino acid) in line 50A, 15 in 51A, 21 in 55A, eight in WT1, and seven in WT2. In the LOW category, the number of synonymous amino acid changes (the main type in this category) was slightly higher in 50A than in the other four lines.

**Table 3:** **Prediction of the effects of variants**

**Impact (percentage in MucoRice-CTBs/WTs)**	**Effect type**	**Number of line-specific classified variants**				
		**50A**	**51A**	**55A**	**WT1**	**WT2**
**HIGH (1.4%/1.2%)**	FRAME_SHIFT	5	8	12	10	5
	SPLICE_SITE_ACCEPTOR	1	0	0	1	0
	SPLICE_SITE_DONOR	2	0	0	0	0
	START_LOST	0	0	0	0	0
	STOP_GAINED	1	0	0	0	0
	STOP_LOST	2	0	0	0	0
**MODERATE (3.0%/1.7%)**	CODON_CHANGE_PLUS_CODON_DELETION	0	1	1	0	2
	CODON_CHANGE_PLUS_CODON_INSERTION	0	0	0	0	0
	CODON_DELETION	2	1	5	1	5
	CODON_INSERTION	0	1	0	0	0
	NON_SYNONYMOUS_CODING	21	15	21	8	7
**LOW (1.4%/1.3%)**	NON_SYNONYMOUS_START	0	0	0	0	0
	SPLICE_SITE_REGION	3	0	0	0	3
	START_GAINED	3	1	0	1	0
	SYNONYMOUS_CODING	13	4	7	7	7
	SYNONYMOUS_STOP	1	0	0	0	0
**MODIFIER (94.2%/95.8%)**	DOWNSTREAM	307	237	337	205	323
	INTERGENIC	395	305	404	250	407
	INTRAGENIC	0	0	0	0	0
	INTRON	30	31	40	29	56
	UPSTREAM	8	6	10	7	4
	UTR_3_PRIME	1	4	2	0	2
	UTR_5_PRIME	3	5	3	5	5

Line-specific variants (SNPs and InDels) that may affect protein function were categorized into 23 types. These types were further grouped into HIGH, MODERATE, LOW, and MODIFIER according to potential severity. The assignment criteria were pre-defined in the annotation program (SNPEff).

## Discussion

In this study, we analyzed three MucoRice-CTB lines and two untransformed (WT) lines, one of which was originally used to produce the MucoRice-CTB lines. Our purpose was to assess, using NGS, the genome-wide effects of the *Agrobacterium*-mediated transformation system used to generate the MucoRice-CTB lines by comparing them to WT lines, and to validate NGS as a useful tool to confirm the inheritance of the transgene over the passage of generations.

The high-throughput Illumina HiSeq2000 platform provided a large number of paired-end short reads of superior quality from all samples. The coverage was >99% relative to the rice reference genome (Table 1). The number of variants (SNPs and InDels) per 500 kbp varied greatly depending on the chromosome and position within the chromosome. Highly condensed repetitive sequences have been found around the centromeres in rice [23] and the biased amplification efficiency during NGS or the increased probability of mapping errors often occur within or around such regions [24]. Our results showed that there were variant-rich regions on chromosomes 1 and 10 in which we detected sharp peaks of variant distribution near the centromeres (Figure 1A). The distribution of the average numbers of variants along the genome was similar in MucoRice-CTB and WT. Despite considerable variation in the variant densities among chromosomes, the differences between MucoRice-CTBs and WTs within the same region were small (at most 16.7 per 500 kbp; Figure 1B). These results suggest that MucoRice-CTBs and WTs have few differences in terms of genome-wide distribution of the number of variants.

Kawakatsu et al. [25] compared the variants in two mutant lines of cultivar Koshihikari: one line was generated by gamma radiation and ethyl methanesulfonate, and the second line was derived from the first one by *Agrobacterium*-mediated transformation. The transformation-specific mutation rate was determined as 5.5 × 10^-7^/site. In our study, MucoRice-CTB-specific variant rates ranged from 1.41 × 10^-6^ to 2.28 × 10^-6^, which are 2–4 times that from Kawakatsu’s report. This difference may be due to the differences in cultivars or data analysis.

For the set of five lines, we analyzed line-specific variants, variants shared by two, three, and four lines, and those shared by all five lines (common variants) (Figure 3); the common variants were the most abundant. No considerable difference was observed in the average numbers of line-specific variants (either total, SNPs, insertions or deletions) between WTs and MucoRice-CTBs (Figure 3). The pattern of nucleotide substitutions was similar and biased towards G to A and C to T in both groups (Figure 2A). This Ts has been reported to be caused by UV-radiation and the deamination of methylated C [26]. The Ts/Tv ratios determined in our study are similar to the Ts/Tv ratio for rice regenerated from long-term cell culture [27]. A higher number of G to T and C to A Tv was found in MucoRice-CTBs than in WTs; this might explain the difference in the Ts/Tv ratio between MucoRice-CTBs (1.12) and WTs (1.65) (Figure 2). The difference in Tv frequencies between MucoRice-CTBs and WT can be explained by observations of Cheng et al. [28], who reported that oxidized G (8-hydroxy-G), which is often detected in living cells, may pair with A instead of C, resulting in a subsequent change of G to T. In our study, sterilization with sodium chlorite prior to MucoRice-CTB callus generation may have caused G oxidation (followed by insufficient repair). Because the transformation system we used consists of several steps including seed sterilization, callus induction, co-transformation with *Agrobacterium*, plant regeneration, and the passage of generations, the specific factor(s) responsible for genome-wide variations (other than oxidation by sodium hypochlorite during seed sterilization) remain to be elucidated.

Most line-specific variants (MucoRice-CTBs: 94.2%, WTs: 95.8%) belonged to the MODIFIER category (Table 3). According to the SNPEff manual [22], this category includes non-coding variants or variants affecting non-coding genes, which are unlikely to have marked effects on protein functions. The number of non-synonymous coding variants was slightly higher in all MucoRice-CTB lines than in WT lines and may have resulted from G oxidation mentioned above, leading to amino acid substitutions.

Since all MucoRice-CTB lines were generated from WT1 seed stock, we expected that variants from the WT1 would be inherited by MucoRice-CTBs. When the variants inherited to the progenies, the comparison of WT1 with MucoRice-CTBs should result in no different. However, line-specific variants were still observed in all lines and their numbers were similar (Figure 3). Some calli were chosen from different seed scutella; WT seeds used for generation of MucoRice-CTB lines and for genomic analysis were from different individuals. Therefore, line-specific variants in MucoRice-CTBs and WTs may be mainly due to individual differences within the same cultivar, and *Agrobacterium*-mediated transformation system may have only a limited effect on the genome.

Recently, seven domesticated and landrace cultivars were resequenced with NGS and compared with the rice reference cultivar Nipponbare. The total numbers of variants in these strains were 168,165 for Omachi, 158,310 for Yamadanishiki, 120,675 for Kameji, 180,402 for Gohyakumangoku, 147,639 for Koshihikari, 109,972 for Norin-8, and 987,045 for Moroberekan [29]. Another study reported 67,000 SNPs detected by NGS in Koshihikari in comparison with the rice reference [17]. In the present study, we used Nipponbare, the same cultivar as in the Rice Genome Project [30]; within each line, we detected ~20,000 total variants and on average 720 line-specific variants (Table 2), which presumably resulted from individual differences in each line. Thus, the numbers of variants between different cultivars appear to be much larger than those between individual lines within the same cultivar.

## Conclusions

We conclude that MucoRice-CTB and WT are almost identical at the genomic level and that the genome-wide effects in marker-free MucoRice-CTB lines were slight in comparison with the individual difference in WT seed stocks. Some difference in the prevalence of nucleotide substitutions between MucoRice-CTBs and WTs may be caused by the *Agrobacterium*-mediated transformation system. It is essential to find and to control the affecting factors. An accurate genome-wide assessment technology enabled by further improvements in NGS platform, in terms of both hardware and software, could become a key approach in manufacturing plant-made pharmaceuticals.

## Methods

### MucoRice-CTB and WT lines used

In a previous study, we established six HPT selection marker–free MucoRice-CTB lines by using two different *A. tumefaciens* strains, each carrying a distinct T-DNA vector for co-transformation [11]. The T-DNA vectors contained either the CTB gene with an RNAi cassette or an HPT selection marker cassette. The two T-DNA vectors were introduced into calli and hygromycin-mediated selection was performed. Segregation of the HPT marker gene from the transformant genomes was achieved by the passage of generations. Marker-free transformants were then propagated for at least five generations obtained by self-pollination to fix the desired transgene. Line 51A of MucoRice-CTB was selected because it had the highest CTB expression as a seed bank for vaccine production for human use; the genomic location and structure of the transgenes were determined in this line [11]. In this study, three out of six selection marker–free MucoRice-CTB lines (50A, 51A, and 55A) and two WT rice lines of the same cultivar (WT1 and WT2) were analyzed by NGS. The WT1 stock was previously used to generate MucoRice-CTB; WT2 was maintained by a commercial seed provider. The removal of the selection marker gene and the presence of the CTB gene in three MucoRice-CTB lines were confirmed by PCR analysis (Additional file 1: Figure S1). Cultivation, including germination, was performed hydroponically in growth chambers (352-PJ, Panasonic, Japan). Approximately three-week-old seedlings were used for genomic DNA extraction.

### PCR analysis

Genomic DNA was isolated from leaves of WT and transgenic plants by using a Nucleon PhytoPure kit (GE Healthcare, Madison, WI, USA). PCR was conducted by using GoTaq Master Mix (Promega, Madison, WI, USA) and a GeneAmp PCR System 9700 (Applied Biosystems, Carlsbad, CA, USA) under the following conditions: 1 min at 94°C; and 35 cycles of 30 s denaturation at 94°C, 30 s annealing at 60°C, and 1 min extension at 72°C. The PCR products were separated by electrophoresis on a 2.0% (w/v) agarose gel. Binary vectors carrying HPT or CTB were used as positive control of the analysis.

### Whole-genome resequencing

Total DNA (~1.0 μg from each line) was fragmented by using a Covaris instrument (Covaris, Woburn, MA, USA). Both ends of the DNA fragments from each line were blunted and phosphorylated; 3′-dA overhangs and index adapters were then attached. Fragments of 250–500 bp (excluding adapter sequences) were selected by agarose gel electrophoresis, and a sequence library was generated by mild PCR amplification of the selected fragments. The quality of the sequence library, in terms of peak fragment size and concentration, was examined by using an Agilent 2100 Bioanalyzer (Agilent Technologies UK Ltd., Berkshire, UK). Sequence clusters on a flow cell were prepared by using a cBot clustering system (Illumina, San Diego, CA, USA). DNA was sequenced with an Illumina HiSeq2000 platform (Illumina). Paired-end read sequences (100 bps per read; Sanger FASTQ format) from both sides of each fragment were obtained with CASAVA software (ver. 1.13.48; Illumina).

Resequencing genomic data of two WT lines were uploaded and submitted in the public repository of DDBJ (DDBJ Sequence Read Archive, DRA, http://www.ddbj.nig.ac.jp/index-e.html) with the accession number of DRA002860.

### Mapping reads to the reference genome

Mapping of the 100-bp short reads to the rice reference genome sequence (Os-Nipponbare–Reference-IRGSP-1.0 build 5) [20,21] was performed using Burrows-Wheeler Aligner (BWA ver. 0.5.9) [31]. The mapping function ‘aln’ of BWA was used to generate intermediate files. These were then used to generate SAM files (which contained mapped read information) by running the ‘sampe’ function. Both algorithms were used with default parameters. The SAM files, which are normally very large, were converted into binary BAM files by using the ‘view’ function of SAMtools [32]. The BAM files were then sorted by using the ‘sort’ function of SAMtools. Duplicate reads in sorted BAM files were removed with Picard tools [33] with the following parameters: REMOVE_DUPLICATES = true, AS = true, SORTING_COLLECTION_SIZE_RATIO = 0.1, and VALIDATION_STRINGENCY = LENIENT. Mapping rate was calculated as the ratio between the numbers of mapped reads and total reads. Coverage rate, which is the ratio between the length of the genomic region covered by at least one read and the length of the reference genome was calculated by identifying all uncovered regions in the genome using the ‘genomeCoverageBed’ function of the BEDTools package [34] with the option ‘-bga’.

### Detecting SNPs and InDels

SNPs and short InDels between the mapped read data and the reference genome were called with SAMtools by using the mpileup function with ‘-uf’ options and default parameters, and then the data format of ‘bcf’ was converted into ‘vcf’ with BCFTools [35]. We then used varFilter in vcfutils (part of the SAMtools package) to remove variants covered by an excessive number of reads (>10,000). Called variants were annotated on the basis of information on gene structure and function from the Rice Annotation Project by using SNPEff (ver. 3.4) [36]. The potential effect of each variant on gene expression and protein structure or function was examined by SNPEff.

### Variant filtration

All variants from the five lines were listed according to their genomic positions; to minimize the number of false-positives, variant filtration was performed according to three criteria: (1) The phred-scaled score (calculated by mpileup in SAMtools) must be at least 30. This criterion guarantees the probability of false positives of ≤0.001. (2) The position of each variant must be covered by at least four reads in each of the five lines regardless of whether the variant was present at the position. Information on the number of reads covering specific positions was obtained by using the coverageBED function in BEDTools [34]. (3) If a variant is shared by more than one line, the alteration type needs to be the same; for example, if an SNP was detected at a certain position in one line whereas an insertion was detected in the same position in another line, these variants were excluded. This criterion was adopted to create the variant-sharing profile, i.e. a ‘shared’ variant needs to be of the same type and be present at the same position.

### Calculation of mutation rates

Mutation rates were calculated by dividing the total number of each line-specific variants by covered length.

## Availability of supporting data

The data sets supporting the results of this article are available in the DDBJ repository, DDBJ Sequence Read Archive (DRA), with the accession number DRA002860 in http://www.ddbj.nig.ac.jp/index-e.html.

## Additional file

Additional file 1: Figure S1. Confirmation of removal of the selection marker (HPT gene) and the presence of the CTB gene in MucoRice-CTB. PCR was performed with primer sets specific for HPT (A) or CTB (B) on genomic DNA from MucoRice-CTB lines (50A, 51A, and 55A), and WT lines (WT1 and WT2). PCR products were analyzed by agarose gel electrophoresis. Arrowheads show the positions of the HPT amplicon (969 bps; A) detected only in positive control (PC; HPT gene-carrying binary vector) and CTB amplicon (312 bp, B) in positive control (CTB gene-carrying binary vector) lane. NC represents negative control. X174/*Hae*III is used as size marker.

## Abbreviations

BWA: Burrows-wheeler aligner
CT: Cholera toxin
CTB: Cholera toxin B-subunit
HPT: Hygromycin phosphotransferase
InDels: Insertions and deletions
NGS: Next generation sequencing/sequencer
PMP: Plant-made pharmaceuticals
RNAi: RNA interference
SNP: Single nucleotide polymorphism
T-DNA: Transfer DNA
Ts: Transitions
Tv: Transversions
WT: Wild-type

## References

[1] Ma JK-C, Barros E, Bock R, Christou P, Dale PJ, Dix PJ (2005). Molecular farming for new drugs and vaccines. Current perspectives on the production of pharmaceuticals in transgenic plants. EMBO Rep.

[2] Twyman R, Stoger E, Schillberg S, Christou P, Fischer R (2003). Molecular farming in plants: host systems and expression technology. Trends Biotechnol.

[3] Sil B, Jha S (2014). Plants: the future pharmaceutical factory. Am J Plant Sci.

[4] Paul M, Ma J (2011). Plant-made pharmaceuticals: leading products and production platforms. Biotechnol Appl Biochem.

[5] Stoger E, Fischer R, Moloney M, Ma J (2014). Plant molecular pharming for the treatment of chronic and infectious diseases. Annu Rev Plant Biol.

[6] Sabalza M, Christou P, Capell T. Recombinant plant-derived pharmaceutical proteins: current technical and economic bottlenecks. Biotechnol Lett. 2014; 36(12):2367–79. 10.1007/s10529-014-1621-3 25048244

[7] Nochi T, Takagi H, Yuki Y, Yang L, Masumura T, Mejima M (2007). Rice-based mucosal vaccine as a global strategy for cold-chain- and needle-free vaccination. Proc Natl Acad Sci U S A.

[8] Nochi T, Yuki Y, Katakai Y, Shibata H, Tokuhara D, Mejima M (2009). A rice-based oral cholera vaccine induces macaque-specific systemic neutralizing antibodies but does not influence pre-existing intestinal immunity. J Immunol.

[9] Tokuhara D, Yuki Y, Nochi T, Kodama T, Mejima M, Kurokawa S (2010). Secretory IgA-mediated protection against V. cholerae and heat-labile enterotoxin-producing enterotoxigenic Escherichia coli by rice-based vaccine. Proc Natl Acad Sci U S A.

[10] Yuki Y, Mejima M, Kurokawa S, Hiroiwa T, Takahashi Y, Tokuhara D (2013). Induction of toxin-specific neutralizing immunity by molecularly uniform rice-based oral cholera toxin B subunit vaccine without plant-associated sugar modification. Plant Biotechnol J.

[11] Mejima M, Kashima K, Kuroda M, Takeyama N, Kurokawa S, Fukuyama Y, et al. Determination of genomic location and structure of the transgenes in marker-free rice-based cholera vaccine by using whole genome resequencing approach. Plant Cell Tiss Org Cult. 2015; 120(1):35–48.

[12] Bao P, Granata S, Castiglione S, Wang G, Giordani C, Cuzzoni E (1996). Evidence for genomic changes in transgenic rice (*Oryza sativa* L.) recovered from protoplasts. Transgenic Res.

[13] Latham J, Wilson A, Steinbrecher R (2006). The mutational consequences of plant transformation. J Biomed Biotechnol.

[14] Davey J, Hohenlohe P, Etter P, Boone J, Catchen J, Blaxter M (2011). Genome-wide genetic marker discovery and genotyping using next-generation sequencing. Nat Rev Genet.

[15] Bräutigam A, Gowik U (2010). What can next generation sequencing do for you? Next generation sequencing as a valuable tool in plant research. Plant Biol (Stuttg).

[16] Nowrousian M (2010). Next-generation sequencing techniques for eukaryotic microorganisms: sequencing-based solutions to biological problems. Eukaryot Cell.

[17] Yamamoto T, Nagasaki H, Yonemaru J, Ebana K, Nakajima M, Shibaya T (2010). Fine definition of the pedigree haplotypes of closely related rice cultivars by means of genome-wide discovery of single-nucleotide polymorphisms. BMC Genomics.

[18] Subbaiyan G, Waters D, Katiyar S, Sadananda A, Vaddadi S, Henry R (2012). Genome-wide DNA polymorphisms in elite indica rice inbreds discovered by whole-genome sequencing. Plant Biotechnol J.

[19] Hu Y, Mao B, Peng Y, Sun Y, Pan Y, Xia Y (2014). Deep re-sequencing of a widely used maintainer line of hybrid rice for discovery of DNA polymorphisms and evaluation of genetic diversity. Mol Genet Genomics.

[20] Sakai H, Lee S, Tanaka T, Numa H, Kim J, Kawahara Y (2013). Rice Annotation Project Database (RAP-DB): an integrative and interactive database for rice genomics. Plant Cell Physiol.

[21] RAP-DB [http://rapdb.dna.affrc.go.jp/download/irgsp1.html]

[22] SNPEff [http://snpeff.sourceforge.net/SnpEff_manual.html]

[23] Cheng Z, Dong F, Langdon T, Ouyang S, Buell C, Gu M (2002). Functional rice centromeres are marked by a satellite repeat and a centromere-specific retrotransposon. Plant Cell.

[24] Treangen T, Salzberg S (2012). Repetitive DNA and next-generation sequencing: computational challenges and solutions. Nat Rev Genet.

[25] Kawakatsu T, Kawahara Y, Itoh T, Takaiwa F (2013). A whole-genome analysis of a transgenic rice seed-based edible vaccine against cedar pollen allergy. DNA Res.

[26] Ossowski S, Schneeberger K, Lucas-Lledó J, Warthmann N, Clark R, Shaw R (2010). The rate and molecular spectrum of spontaneous mutations in *Arabidopsis thaliana*. Science.

[27] Miyao A, Nakagome M, Ohnuma T, Yamagata H, Kanamori H, Katayose Y (2012). Molecular spectrum of somaclonal variation in regenerated rice revealed by whole-genome sequencing. Plant Cell Physiol.

[28] Cheng K, Cahill D, Kasai H, Nishimura S, Loeb L (1992). 8-Hydroxyguanine, an abundant form of oxidative DNA damage, causes G → T and A → C substitutions. J Biol Chem.

[29] Arai-Kichise Y, Shiwa Y, Nagasaki H, Ebana K, Yoshikawa H, Yano M (2011). Discovery of genome-wide DNA polymorphisms in a landrace cultivar of Japonica rice by whole-genome sequencing. Plant Cell Physiol.

[30] Goff S, Ricke D, Lan T, Presting G, Wang R, Dunn M (2002). A draft sequence of the rice genome (*Oryza sativa* L. ssp. *japonica*). Science.

[31] Li H, Durbin R (2009). Fast and accurate short read alignment with Burrows–Wheeler transform. Bioinformatics.

[32] Li H, Handsaker B, Wysoker A, Fennell T, Ruan J, Homer N (2009). The Sequence Alignment/Map format and SAMtools. Bioinformatics.

[33] Picard tools [http://broadinstitute.github.io/picard/]

[34] Quinlan A, Hall I (2010). BEDTools: a flexible suite of utilities for comparing genomic features. Bioinformatics.

[35] BCFTools man page: [http://samtools.github.io/bcftools/bcftools.html]

[36] Cingolani P, Platts A, Wang L, Coon M, Nguyen T, Wang L (2012). A program for annotating and predicting the effects of single nucleotide polymorphisms, SnpEff: SNPs in the genome of *Drosophila melanogaster* strain w^1118^; iso-2; iso-3. Fly (Austin).

