# Supplementary figures and images for: Comparative whole-genome analyses of selection marker–free rice-based cholera toxin B-subunit vaccine lines and wild-type lines
Source: BMC Genomics. 2015 Feb 5;16(1):48. doi: 10.1186/s12864-015-1285-y (PMC4320824; doi:10.1186/s12864-015-1285-y)

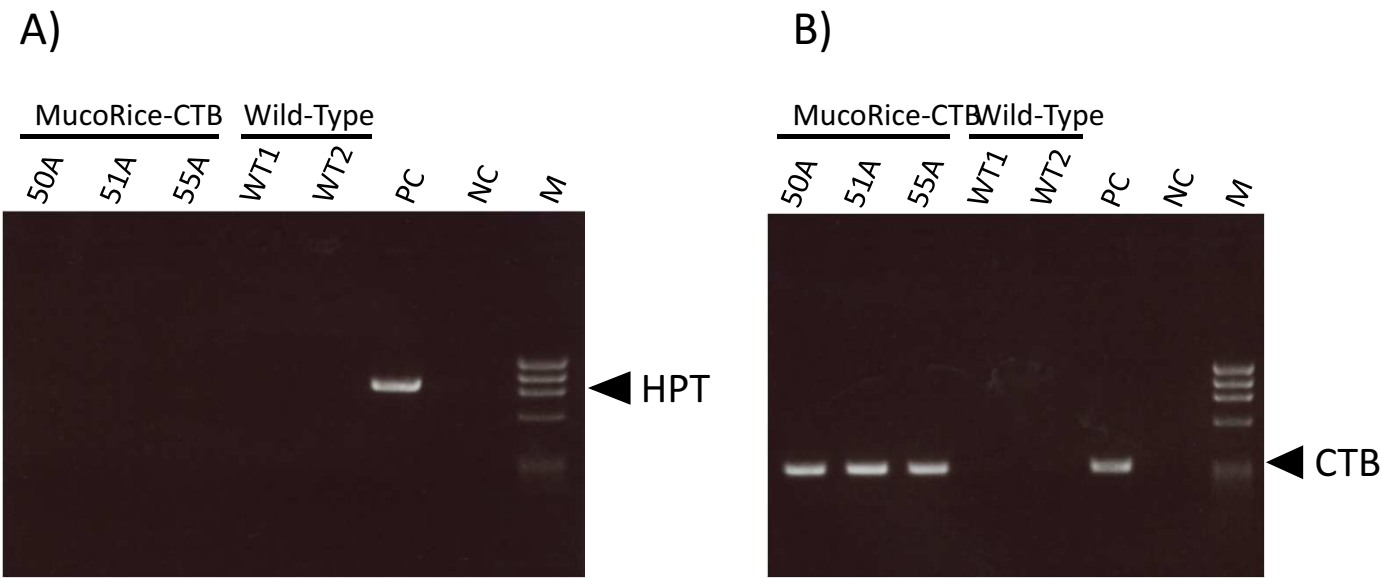

Supplement: Additional file 1: Figure S1. — Confirmation of removal of the selection marker (HPT gene) and the presence of the CTB gene in MucoRice-CTB. PCR was performed with primer sets specific for HPT (A) or CTB (B) on genomic DNA from MucoRice-CTB lines (50A, 51A, and 55A), and WT lines (WT1 and WT2). PCR products were analyzed by agarose gel electrophoresis. Arrowheads show the positions of the HPT amplicon (969 bps; A) detected only in positive control (PC; HPT gene-carrying binary vector) and CTB amplicon (312 bp, B) in positive control (CTB gene-carrying binary vector) lane. NC represents negative control. X174/HaeIII is used as size marker. [file 12864_2015_1285_MOESM1_ESM.pdf]
